# In‐Hospital Complications of Influenza A and B Among Hospitalized Australian Children in the Post‐COVID Era

**DOI:** 10.1111/crj.70217

**Published:** 2026-07-23

**Authors:** Angelica Wei Wen Hii, Jahidur Rahman Khan, Kishor Kumar Paul, Mei Chan, Nan Hu, Brendan McMullan, Philip N. Britton, Adam W. Bartlett, Rama Kandasamy, Louisa Owens, Bernadette Prentice, Adam Jaffe, Nusrat Homaira

**Affiliations:** ^1^ Discipline of Paediatrics and Child Health, School of Clinical Medicine University of New South Wales Sydney Australia; ^2^ School of Population Health University of New South Wales Sydney Australia; ^3^ Department of Respiratory Medicine Sydney Children's Hospital, Randwick Sydney Australia; ^4^ Sydney Medical School and Sydney Infectious Diseases University of Sydney Sydney Australia; ^5^ National Centre for Immunization Research and Surveillance The Children's Hospital at Westmead Sydney Australia; ^6^ Sydney Medical School University of Sydney Sydney Australia; ^7^ Children's Health Ireland Crumlin Ireland

**Keywords:** children, hospitalization, influenza, in‐hospital complications, post‐COVID‐19

## Abstract

**Background:**

Influenza is a leading cause of pediatric hospitalizations, with clinical presentations and outcomes varying between types A and B. This study compared the clinical characteristics and complications of children hospitalized with influenza A and B at the Sydney Children's Hospitals Network (SCHN) in the post‐COVID era.

**Methods:**

We conducted a retrospective cohort study of children < 18 years admitted to SCHN with influenza during 2022–2023. Data on patient demographics, clinical presentations, complications, and management were extracted from electronic medical records. Group comparisons used chi‐square or Fisher's exact tests, and modified Poisson regression estimated adjusted risk ratios (RRs) with 95% confidence intervals (CIs) for key clinical outcomes.

**Results:**

Among 704 influenza hospitalizations, 480 (68.2%) were due to influenza A and 224 (31.8%) to influenza B. Influenza B hospitalizations were more frequent in older children aged 5–< 18 years (67.4% vs. 57.7%) and associated with myalgia and pharyngitis, whereas influenza A hospitalizations were more common in children < 5 years (42.3% vs. 32.6%) and linked to cough, wheeze, and seizures. Although less common, influenza B hospitalizations exhibited greater clinical severity, with higher risks of noninvasive respiratory support (RR = 2.94; 95% CI 1.49–5.81) and intensive care unit (ICU) admission and/or mechanical ventilation (RR = 3.23; 95% CI 1.43–7.32).

**Conclusions:**

Influenza B, though less prevalent, was associated with greater severity, particularly in older children. These findings underscore the importance of continued protection against both influenza types and support consideration of expanding vaccine access to children aged 5 years and older.

## Graphical abstract

In the post‐COVID‐19 Australian pediatric cohort of 704 hospitalizations, influenza B accounted for one‐third of cases but was associated with greater severity than influenza A. Children with influenza B had higher risks of noninvasive respiratory support and intensive care unit admission and/or mechanical ventilation, particularly among those aged 5–18 years. These findings support continued vaccination against both influenza types.

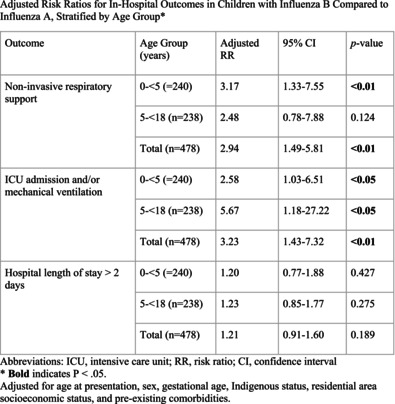

## 1 Introduction

Influenza viruses are a major cause of respiratory illness globally, responsible for an estimated 9.5 million lower respiratory infection hospitalizations annually, with the highest burden among children under 10 years [1]. In Australia, influenza is a leading contributor to pediatric hospital admissions and healthcare costs, reaching AUD 3.247 billion in 2022–2023, largely due to public hospitalizations [2].

Human influenza viruses are classified as Types A, B, and C, with Type A causing seasonal epidemics and occasional pandemics, Type B causing periodic large epidemics but not pandemics, and Type C typically being endemic and causing sporadic, mild respiratory disease [3]. Influenza A is divided into subtypes based on hemagglutinin and neuraminidase glycoproteins, whereas influenza B circulates as two antigenically distinct lineages, B/Victoria and B/Yamagata [4]. Although influenza B has historically been considered less severe than influenza A, its clinical impact in children remains incompletely understood, and emerging evidence suggests comparable or, in some settings, greater severity [5].

In children, risk factors for severe influenza include young age [6, 7, 8, 9], sex differences before and after puberty [6, 10], prematurity, preexisting comorbidities [8, 9], and socioeconomic disadvantage [11].

Clinical presentations of influenza A and B are broadly similar, commonly including fever, respiratory symptoms, headache, and gastrointestinal manifestations [12, 13, 14]. Influenza B more often affects older children and is associated with pharyngitis and myalgia [15, 16, 17], whereas influenza A predominates in younger children [14]. Despite these trends, both viruses present with clinically indistinguishable features, with most studies reporting no meaningful differences in symptoms or outcomes [12, 14, 15, 16, 18, 19, 20]. Influenza complications include pneumonia, otitis media, sinusitis, myositis, and, in severe cases, neurological or cardiovascular involvement [18, 21]. Several studies have compared influenza A and B in children with inconsistent findings [19, 20, 22]. Evidence on subtype‐specific severity remains mixed, with most pre‐pandemic studies reporting no significant differences in outcomes between influenza A and B, whereas others suggest increased severity associated with influenza B in certain age groups, though some suggest greater influenza B severity in older children [14, 18, 19, 20, 22, 23]. Historically, research emphasis on influenza A may have underestimated the burden of influenza B [24].

Vaccination remains the cornerstone of influenza prevention. In Australia, the National Immunization Program funds quadrivalent influenza vaccines for children aged 6 months to < 5 years and other high‐risk groups [25]. However, vaccination coverage among Australian children remains suboptimal [26], and uptake varies by age. These differences in coverage are important when interpreting subtype‐specific disease severity.

The COVID‐19 pandemic substantially disrupted the circulation of influenza viruses, with near absence of seasonal activity followed by atypical resurgence patterns after the relaxation of public health measures [27]. These disruptions likely altered population immunity, particularly among young children, and were accompanied by changes in healthcare‐seeking behavior, hospital admission thresholds, and diagnostic testing practices. As a result, the epidemiology and clinical severity of influenza observed in the post‐pandemic period may differ from pre‐pandemic patterns, limiting the generalisability of historical comparisons between influenza A and B.

Despite renewed influenza circulation in the post‐COVID period, there is limited contemporary evidence describing the relative clinical impact of influenza A and B among hospitalized children. This study therefore aimed to describe the clinical characteristics and in‐hospital outcomes of children hospitalized with laboratory‐confirmed influenza A and B during 2022–2023 and to examine subtype‐associated differences in severity within a post‐pandemic healthcare context.

## 2 Methods

### 2.1 Study Design and Setting

We conducted a retrospective study comparing clinical characteristics and in‐hospital outcomes of children hospitalized with laboratory‐confirmed influenza A and B. The study included all episodes of laboratory‐confirmed influenza in children < 18 years admitted to Sydney Children's Hospitals Network (SCHN) from January 2022 to December 2023 (two influenza seasons). SCHN comprises the Sydney Children's Hospital, Randwick, and the Children's Hospital at Westmead, the two largest pediatric hospitals in New South Wales (NSW).

### 2.2 Study Population

Cases were included if influenza A or B infection was confirmed by polymerase chain reaction (PCR) testing at admission or during hospitalization and if the child had an acute respiratory infection, defined by upper or lower respiratory symptoms (e.g., cough, coryza, sore throat, shortness of breath, wheeze, or tachypnea), with or without fever where a laboratory testing of respiratory specimen (nasopharyngeal aspirate or nose and throat swab) was done on admission and was positive for either influenza A or influenza B. Children presenting to the emergency department with laboratory‐confirmed influenza and a hospital stay of at least 24 h were also included, excluding brief stays lasting only a few hours. Because COVID‐19 pandemic all children hospitalized with ALRI at SCHN are routinely tested for a panel of respiratory pathogens using multiplex respiratory viral PCR panels detecting respiratory syncytial virus, adenovirus, bocavirus, coronavirus, parainfluenza, enterovirus, rhinovirus, and human metapneumovirus. Children with co‐detection of both influenza types or laboratory evidence of co‐infections of either of the influenza types with other respiratory viral pathogen including respiratory syncytial virus, human metapneumovirus, parainfluenza virus, rhinovirus, coronaviruses, and adenovirus were excluded.

### 2.3 Data Collection

Data were extracted from the electronic medical record (eMR) system with support from the SCHN Health Information Unit. Extracted variables included demographic characteristics, clinical presentation, investigations, management, and outcomes.

### 2.4 Exposure Variables

Demographic data at admission included sex, age (years), country of birth (Australia/overseas), Indigenous status (yes/no), primary language spoken at home (English/other), and residential postcode. Socioeconomic status was assessed using the Socio‐Economic Indexes for Areas (SEIFA) Index of Relative Socioeconomic Advantage and Disadvantage (IRSAD) based on residential postcode from the 2021 Australian Census [28]. IRSAD ranks geographic areas by relative socioeconomic conditions and groups them into quartiles. These quartiles represent area‐level advantage or disadvantage and are based on the number of areas rather than population size. The first quartile (0–25th percentiles) represents the most disadvantaged areas, and the fourth quartile (76th–100th percentiles) represents the least disadvantaged.

As influenza vaccination is recommended and fully funded for children aged 6 months to < 5 years under the NIP, age was categorized as 0–< 5 and 5–< 18 years. Additional data from clinical notes included gestational age (preterm defined as < 37 weeks) and preexisting comorbidities (e.g., chronic pulmonary, cardiac, endocrine, hepatic, renal, or neurological disorders; genetic or chromosomal conditions; anatomical abnormalities; malignancies; and immunosuppressive conditions).

### 2.5 Clinical Presentation and Definitions

Symptoms at presentation were extracted from clinical notes and included apnea, central cyanosis, chest pain, conjunctivitis, cough, crackles, dyspnea, fever, gastrointestinal symptoms (abdominal pain, diarrhea, nausea, or vomiting), headache, hypoxia, lethargy, nasal symptoms (rhinorrhea or nasal congestion), pharyngitis, reduced oral intake, seizures, severe respiratory distress (grunting, nasal flaring, tracheal tug, or chest indrawing), tachypnea, and wheeze. Fever, tachypnea, and hypoxia were defined according to the NSW Health Standard Pediatric Observations Chart: fever as temperature > 38°C, hypoxia as SpO_2_ < 92%, and tachypnea as a respiratory rate of ≥ 60 breaths/min for infants ≤ 2 months, ≥ 50 for > 2–12 months, ≥ 40 for > 1–5 years, ≥ 30 for > 5–12 years, and ≥ 25 for > 12–18 years.

### 2.6 Clinical Outcomes

Outcomes included length of stay (LOS), intensive care unit (ICU) admission, and respiratory support. LOS, expressed in bed‐days, included readmissions within 48 h and was classified as extended if it exceeded the median (2 days). Respiratory support was categorized as noninvasive (high‐flow nasal prongs, continuous or bilevel positive airway pressure) or invasive (mechanical ventilation).

Chest imaging findings, including radiographic consolidation, were recorded based on radiology reports. Secondary bacterial infections were identified through clinical documentation and laboratory findings and included pneumonia, otitis media, bacterial pharyngitis/tonsillitis, tracheitis, periorbital cellulitis, and bacteremia. Pneumonia was diagnosed based on clinical documentation and radiographic evidence; culture confirmation was not available for all cases. Treatment variables included antibiotic and antiviral (oseltamivir) use, as well as non‐oral hydration.

### 2.7 Data Analysis

Continuous variables were summarized as medians with interquartile ranges (IQR) and categorical variables as frequencies with percentages. Group comparisons for categorical variables between influenza A and B were conducted using Pearson's chi‐square or Fisher's exact tests (for small cell counts). Independent *t*‐tests were applied to normally distributed continuous data, and the Mann–Whitney *U* test was applied to non‐normally distributed continuous data.

Modified Poisson regression models, stratified by age group (0–< 5 and 5–< 18 years), were used to estimate adjusted risk ratios (aRRs) with 95% confidence intervals (CIs) for three key outcomes comparing influenza B to A: need for noninvasive respiratory support (yes/no), ICU admission and/or mechanical ventilation (yes/no), and extended hospital LOS (> 2 days; yes/no). Models were adjusted for clinically relevant covariates: age, sex, gestational age, Indigenous status, socioeconomic quartile, and preexisting comorbidities. Age‐stratified analyses were conducted to explore potential effect modification by age.

## 3 Results

### 3.1 Demographic Characteristics

From January 2022 to December 2023, 1560 pediatric hospitalizations with laboratory‐confirmed influenza and other respiratory viral co‐detections were identified. Among these, 704 hospitalizations were positive for influenza A or B alone, including 480 (68.2%) with influenza A and 224 (31.8%) with influenza B. There were five hospitalizations with influenza A and B co‐infection that were excluded from subtype‐specific analyses (Table 1). Sex distribution was similar across both groups, with males comprising approximately 56% of cases. Hospitalizations for influenza B occurred in older children, with a median (IQR) age of 7.6 (3.7–10.0) years versus 5.8 (2.5–9.1) years for influenza A. Most admissions for both influenza A (60.9%) and influenza B (51.6%) were from the least disadvantaged IRSAD quartile. Comorbidities were more common in influenza A hospitalizations than influenza B (47.3% vs. 38.4%; *p* = 0.027) (Table 2).

**TABLE 1:** Distribution of influenza virus types and subtypes among hospitalizations for influenza in children < 18 years at Sydney Children's Hospitals Network, 2022–2023.

Influenza type	Influenza subtype	Age group		
		0–< 5 years	5–< 18 years	All age groups
		*N* (%)	*N* (%)	*N* (%)
Influenza A	H1N1pdm09	53 (19.2)	86 (20.1)	139 (19.7)
	H3N2	37 (13.4)	74 (17.3)	111 (15.8)
	Not subtyped	113 (40.9)	117 (27.3)	230 (32.7)
	Total	203 (73.6)	277 (64.7)	480 (68.2)
Influenza B	Total	73 (26.4)	151 (35.3)	224 (31.8)

**TABLE 2:** Baseline demographic characteristics of children < 18 years hospitalized with influenza A and influenza B at Sydney Children's Hospital Network, 2022–2023.

	Hospitalizations, *N* (%)		*p*
	*N* = 704		
	Influenza A	Influenza B	
Overall	480 (68.2)	224 (31.8)	
Sex: Male	272 (56.7)	124 (55.4)	0.744
Age at presentation (years)			
Median (IQR)	5.8 (6.57)	7.6 (6.26)	**0.002**
Age groups			**0.014**
0–< 5 years	203 (42.3)	73 (32.6)	
5–< 18 years	277 (57.7)	151 (67.4)	
Country of birth			0.809
Australia	446 (92.9)	207 (92.4)	
Overseas	34 (7.1)	17 (7.6)	
Primary language spoken at home			0.373
English	401 (83.5)	193 (86.2)	
Other	79 (16.5)	31 (13.8)	
Residential area socioeconomic status (quartile)			**0.028**
1st (most disadvantaged)	50 (10.5)	37 (16.7)	
2nd	64 (13.4)	39 (17.6)	
3rd	73 (15.3)	31 (14)	
4th (least disadvantaged)	291 (60.9)	114 (51.6)	
Preterm birth^a^			0.086
Yes	46 (9.6)	12 (5.4)	
No	290 (60.4)	135 (60.3)	
Unknown	144 (30)	77 (34.4)	
Any preexisting comorbidities			**0.027**
Yes	227 (47.3)	86 (38.4)	
No	253 (52.7)	138 (61.6)	

*Note:* Data are presented as *n* (column %) unless otherwise specified. Bold indicates *p* < 0.05.

Abbreviation: IQR, interquartile range.

^a^ Preterm birth is defined as a gestational age of less than 37 weeks.

### 3.2 Seasonal Patterns

**FIGURE 1:**
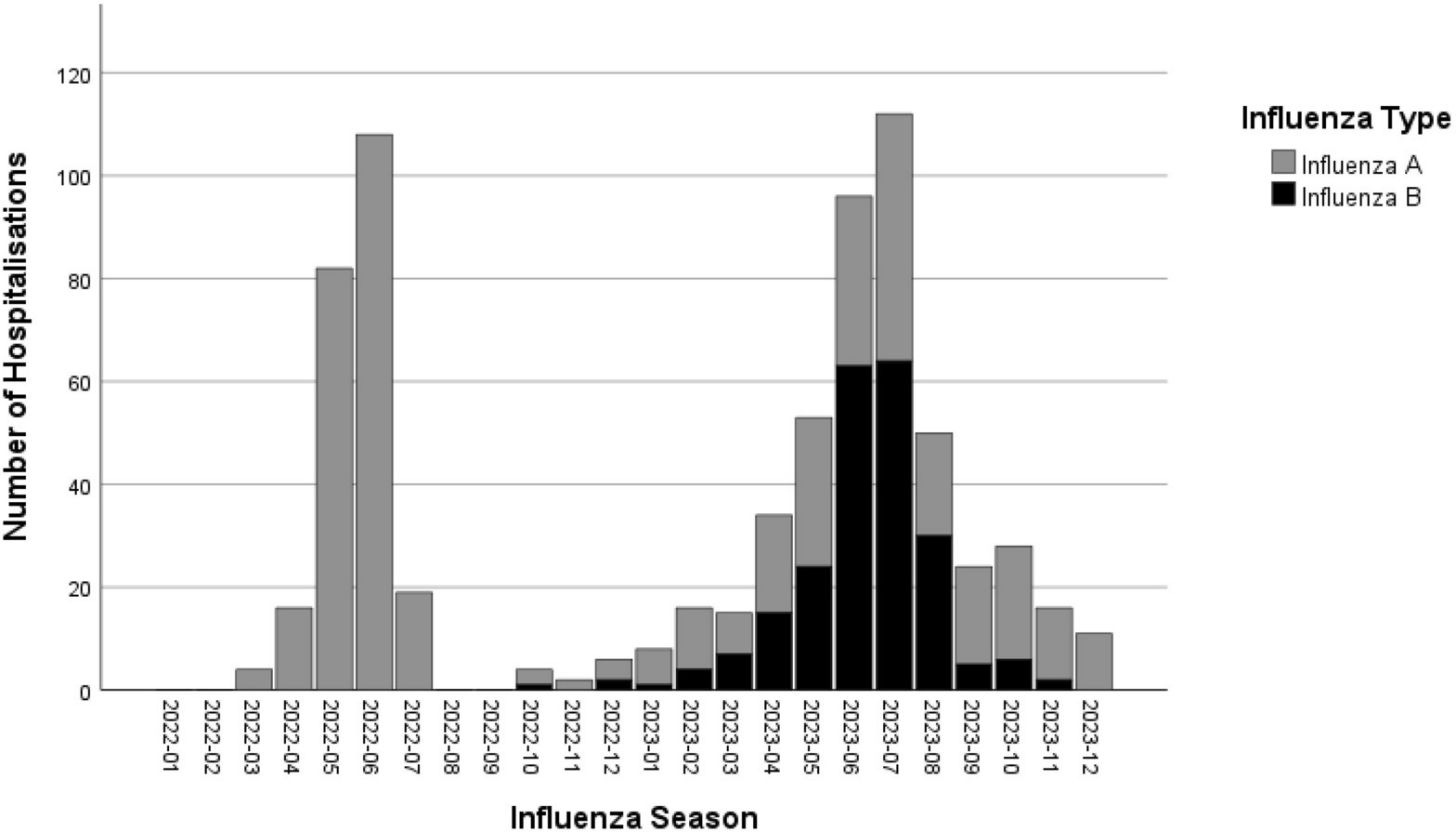
Monthly distribution of influenza A and B hospitalizations among children < 18 years at Sydney Children's Hospitals Network, 2022–2023.

Influenza B hospitalizations in NSW were largely absent in 2022, appearing sporadically from October, whereas influenza A circulated steadily throughout the year, peaking in June. In 2023, influenza B activity increased substantially and exceeded influenza A from June through August (Figure 1).

### 3.3 Clinical Presentation

Overall symptoms were similar between influenza types, including fever, cough, nasal congestion or rhinorrhea, reduced oral intake, and gastrointestinal symptoms. Notable differences included higher rates of myalgia (26.3% vs. 15.4%, *p* < 0.001) and pharyngitis (41.1% vs. 27.3%, *p* < 0.001) in influenza B hospitalizations, versus higher rates of cough (86.3% vs. 77.2%, *p* = 0.003), seizures (7.3% vs. 2.2%, *p* = 0.007), and wheezing (14.8% vs. 8.5%, *p* = 0.020) in influenza A hospitalizations (Table S1).

In children < 5 years, pharyngitis was more frequent in influenza B hospitalizations (34.2% vs. 10.8%, *p* < 0.001). Among those 5–< 18 years, influenza A hospitalizations had higher rates of cough (85.2% vs. 75.5%, *p* = 0.013), seizures (4.7% vs. 0.7%, *p* = 0.024), and wheezing (12.6% vs. 6.0%, *p* = 0.030), whereas influenza B hospitalizations were more often associated with myalgia (35.8% vs. 22.7%, *p* = 0.004) and severe respiratory distress (12.6% vs. 5.4%, *p* = 0.009).

### 3.4 Clinical Outcomes

Median hospital stay was 2 (IQR: 1–3) days for both groups, with approximately one‐third of hospitalizations lasting > 2 days (Table 3). Influenza B hospitalizations had higher rates of noninvasive respiratory support (11.2% vs. 6.0%, *p* = 0.017), mechanical ventilation (6.7% vs. 2.5%, *p* = 0.007), non‐oral hydration (79.5% vs. 66.0%, *p* < 0.001), radiographic consolidation (38.4% vs. 24.3%, *p* = 0.017), and secondary bacterial infection (22.8% vs. 13.8%, *p* = 0.003).

**TABLE 3:** Clinical outcomes by age group and influenza type among children < 18 years hospitalized with influenza A or B at Sydney Children's Hospital Network, 2022–2023.

Clinical outcomes	Age group								
	0–< 5 years		*p*	5–< 18 years		*p*	All age groups		*p*
	Influenza A (*n* = 203)	Influenza B (*n* = 73)		Influenza A (*n* = 277)	Influenza B (*n* = 151)		Influenza A (*n* = 480)	Influenza B (*n* = 224)	
Antibiotic treatment	90 (44.3)	34 (46.6)	0.741	151 (54.5)	79 (52.3)	0.663	241 (50.2)	113 (50.4)	0.953
Antiviral treatment	75 (36.9)	27 (37.0)	0.995	106 (38.3)	64 (42.4)	0.406	181 (37.7)	91 (40.6)	0.459
Non‐oral hydration	128 (63.1)	62 (84.9)	< 0.001	189 (68.2)	116 (76.8)	0.061	317 (66.0)	178 (79.5)	< 0.001
Length of stay, day, median (IQR)	2 (1–3)	2 (1–3)	0.957	2 (1–3)	2 (1–3)	0.827	2 (1–3)	2 (1–3)	0.956
Short stay (≤ 2 days)	148 (72.9)	52 (71.2)	0.784	184 (66.4)	99 (65.6)	0.857	332 (69.2)	151 (67.4)	0.64
Long stay (> 2 days)	55 (27.1)	21 (28.8)		93 (33.6)	52 (34.4)		148 (30.8)	73 (32.6)	
ICU admission	10 (4.9)	9 (12.3)	0.032	16 (5.8)	11 (7.3)	0.54	26 (5.4)	20 (8.9)	0.079
Hours in ICU, median (IQR)	70 (31–191)	65 (41–188)	0.902	33.5 (23–284)	186 (54–439)	0.159	41.5 (24–218)	161.5 (44–324)	0.163
Mechanical ventilation	5 (2.5)	5 (6.8)	0.085	7 (2.5)	10 (6.6)	0.038	12 (2.5)	15 (6.7)	0.007
Hours requiring mechanical ventilation, median (IQR)	47 (17–213)	159 (73–224)	0.347	156 (20–248)	175.5 (133–245)	0.494	123 (20–230)	166 (124–237)	0.196
Any noninvasive respiratory support^a^	13 (6.4)	10 (13.7)	0.053	16 (5.8)	15 (9.9)	0.113	29 (6.0)	25 (11.2)	0.017
Chest radiograph performed	79 (38.9)	31 (42.5)	0.595	106 (38.3)	55 (36.4)	0.707	185 (38.5)	86 (38.4)	0.97
Consolidation detected on chest radiograph^b^	22 (27.8)	11 (35.5)	0.432	23 (21.7)	22 (40)	0.014	45 (24.3)	33 (38.4)	0.017
Any secondary bacterial infection	32 (15.8)	20 (27.4)	0.029	34 (12.3)	31 (20.5)	0.023	66 (13.8)	51 (22.8)	0.003

*Note:* Data are presented as *n* (column %) unless otherwise specified. Bold indicates *p* < 0.05.

Abbreviations: ICU, intensive care unit; IQR: interquartile range.

^a^ Noninvasive respiratory support includes high‐flow nasal prongs, continuous positive airway pressure, and bilevel positive airway pressure.

^b^ Proportions for consolidation on chest radiograph were calculated only among children who had a chest radiograph performed.

In children < 5 years, a higher proportion of influenza B hospitalizations involved ICU admission (12.3% vs. 4.9%, *p* = 0.032), non‐oral hydration (84.9% vs. 63.1%, *p* < 0.001), and secondary bacterial infection (27.4% vs. 15.8%, *p* = 0.029). Among those 5–< 18 years, influenza B had higher rates of mechanical ventilation (6.6% vs. 2.5%, *p* = 0.038), radiographic consolidation (40.0% vs. 21.7%, *p* = 0.014), and secondary bacterial infection (20.5% vs. 12.3%, *p* = 0.023).

Antibiotic use was high across both age groups in influenza A (50.2%) and B (50.4%) hospitalizations, yet confirmed bacterial co‐infection was present in only 13.8% of influenza A and 22.8% of influenza B hospitalizations. Pneumonia accounted for 75 of 117 secondary bacterial infections (64.1%).

In adjusted age‐stratified analyses, influenza B hospitalizations showed higher risk of noninvasive respiratory support (aRR = 2.94; 95% CI 1.49–5.81), particularly among children < 5 years (aRR = 3.17; 95% CI 1.33–7.55), and greater risk of ICU admission and/or mechanical ventilation (aRR = 3.23; 95% CI 1.43–7.32) across both age groups (Table 4).

**TABLE 4:** Adjusted risk ratios for in‐hospital outcomes in children with influenza B compared to influenza A, stratified by age group.

Outcome	Age group (years)	Adjusted RR	95% CI	*p*
Noninvasive respiratory support	0– < 5 (= 240)	3.17	1.33–7.55	**< 0.01**
	5– < 18 (*n* = 238)	2.48	0.78–7.88	0.124
	Total (*n* = 478)	2.94	1.49–5.81	**< 0.01**
ICU admission and/or mechanical ventilation	0– < 5 (= 240)	2.58	1.03–6.51	**< 0.05**
	5– < 18 (*n* = 238)	5.67	1.18–27.22	**< 0.05**
	Total (*n* = 478)	3.23	1.43–7.32	**< 0.01**
Hospital length of stay > 2 days	0– < 5 (= 240)	1.2	0.77–1.88	0.427
	5– < 18 (*n* = 238)	1.23	0.85–1.77	0.275
	Total (*n* = 478)	1.21	0.91–1.60	0.189

*Note:* Adjusted for age at presentation, sex, gestational age, Indigenous status, residential area socioeconomic status, and preexisting comorbidities. Bold indicates *p* < 0.05.

Abbreviations: CI, confidence interval; ICU, intensive care unit; RR, risk ratio.

## 4 Discussion

In this retrospective cohort study of children hospitalized with laboratory‐confirmed influenza during 2022–2023, we observed that influenza B, although less common, was associated with higher markers of in‐hospital severity, including increased use of noninvasive respiratory support and higher rates of ICU admission and mechanical ventilation. These findings provide important insight into the clinical epidemiology of influenza in the post‐COVID period, when viral circulation, population immunity, and healthcare practices have been substantially altered.

In our study, influenza A predominated early in 2022 following the easing of COVID‐19 restrictions, peaking unusually in June (Australian winter), whereas influenza B emerged later, with hospitalizations rising substantially in 2023. In contrast, pre‐pandemic influenza seasons in Australia typically exhibited consistent winter seasonality, with peaks between July and September. The earlier onset and altered timing observed in 2022 and 2023 reflect disrupted circulation patterns consistent with global post‐pandemic shifts [27, 29].

Influenza A hospitalizations were more frequent, consistent with national surveillance data [30, 31] and reflecting the typical annual circulation of influenza A viruses and their greater propensity for antigenic drift [4]. Children admitted with influenza A more often had preexisting comorbidities, supporting evidence that underlying conditions increase disease severity [8, 9], potentially also contributing to higher hospitalization rates. Despite fewer comorbidities, influenza B hospitalizations showed greater clinical severity, suggesting that influenza B may be intrinsically more pathogenic in otherwise healthy children. This may relate to differences in host immune responses or viral factors; notably, influenza B has been associated with higher ICU admission rates compared with influenza A [5].

Unexpectedly, hospitalizations were more common among children from the least disadvantaged quartile, contrasting with literature linking socioeconomic disadvantage to poorer outcomes [11], possibly reflecting catchment demographics, admission practices, health‐seeking behaviors, or unmeasured differences in vaccination uptake, which could not be assessed due to unavailable vaccination data.

The median age of hospitalization exceeded 5 years for both influenza types, with influenza B affecting older children. This aligns with evidence that influenza A predominates in younger children, whereas influenza B disproportionately affects school‐aged children [14, 32, 33]. Notably, children ≥ 5 years not classified as high risk are excluded from the NIP [25], possibly lowering vaccine uptake and contributing to greater severity. Given their role in school‐based transmission and community spread [18], these findings may support extending funded vaccination beyond 5 years and improving public awareness of vaccine benefits.

Clinical presentations overlapped between influenza types, typically including fever, reduced oral intake, respiratory, and gastrointestinal symptoms. Influenza A hospitalizations more commonly involved respiratory symptoms and neurological complications, consistent with previous studies [14, 21, 34]. Conversely, influenza B was more frequently associated with myalgia and pharyngitis, aligning with earlier findings [15, 16, 20, 22]. Older children reported more symptoms, reflecting both increased severity and likely a greater capacity to articulate subjective experiences. Although some clinical features may suggest one subtype, the substantial overlap reinforces the need for laboratory confirmation to distinguish influenza types.

Although national guidelines recommend early antiviral therapy [35], only approximately 40% of hospitalized cases received oseltamivir. Its effectiveness is greatest within 48 h of symptom onset [36], but the timing of initiation could not be determined due to incomplete eMR documentation. Delayed diagnosis, often driven by the nonspecific presentation of influenza resembling other respiratory infections [37], may result in missed treatment windows. Furthermore, a recent US survey found that fewer than half of physicians consistently followed American Academy of Pediatrics antiviral guidelines, reflecting gaps in awareness and adoption [38]. Together, these factors contribute to suboptimal antiviral use and emphasize the importance of prompt testing and timely initiation of treatment when influenza is suspected.

Antibiotic use was high, with nearly half of children receiving antibiotics, regardless of virus type, despite confirmed bacterial co‐infections in only one‐third of treated cases. Although empiric use is common [39], this raises antimicrobial stewardship concerns, as WHO recommendations against routine use in non‐severe influenza without evidence of bacterial co‐infection [40]. The introduction of rapid point‐of‐care influenza testing, now common post‐COVID‐19, could help reduce inappropriate antibiotic use [41] by facilitating timely diagnosis and earlier antiviral initiation [42].

Markers of severity differed between influenza types. Influenza B hospitalizations more often required non‐oral hydration, with 80% of cases receiving intravenous fluids, a finding that suggests greater clinical severity. Dehydration, a recognized marker of illness severity in pediatric respiratory infections [43], often results from inadequate oral intake, vomiting, or diarrhea [44], symptoms common in our cohort. Influenza B hospitalizations were also associated with higher rates of ICU admission, radiographic consolidation, secondary bacterial infection, and respiratory support. These findings align with previous studies reporting comparable [12, 14, 16, 18, 19] or greater severity [7, 22, 23] of influenza B in children and challenge assumptions that influenza A is more virulent. However, heterogeneity across studies in study design, populations, and outcome definitions highlights the need for additional post‐pandemic data to confirm whether influenza B is truly more severe.

Increasing severity with age has also been previously reported [13, 45], consistent with our finding that influenza B predominated in older children and was associated with more severe outcomes. This may partly reflect a lower admission threshold for younger children, leading to the inclusion of milder cases, potential admission bias, and differences in vaccination uptake, although vaccination status was unavailable. Nonetheless, recognizing influenza B as a potentially severe infection in older children remains important for timely management and prevention.

Influenza vaccination coverage among Australian children remains suboptimal and has not returned to pre‐pandemic levels. Among children aged 6 months–< 5 years, coverage fell from 46.1% in 2020 to 26.5% in 2021, recovering only to 34.1% in 2022 and 30.3% in 2023 [26]. Coverage in children aged 5–< 15 years was even lower, dropping from 27.4% in 2020 to 14.5% in 2021, fluctuating at 23.2% in 2022 and 16.4% in 2023 [26]. This is concerning given demonstrated vaccine effectiveness of 44% against hospitalization in 2022 and 68% in 2023, with higher protection against influenza B [30, 31]. Given influenza B's disproportionate impact, continued use of quadrivalent vaccines remains important. Although Australia is considering reverting to trivalent vaccines due to the apparent disappearance of the B/Yamagata lineage [46], ongoing virological surveillance is essential to confirm its absence. Both vaccine types remain effective if matched to circulating strains [46, 47].

This study has several limitations. We did not have pre‐pandemic data that limit our ability to infer whether the severity of influenza B has changed over time. The age distribution of our cohort may also introduce admission bias. Younger children, who were more frequently hospitalized with influenza A, often have lower thresholds for admission due to clinical caution. In contrast, older children with influenza B may represent a more selectively severe group. This difference in admission thresholds could contribute to the higher observed rates of ICU admission and respiratory support in influenza B cases. Although eMR data enabled comprehensive capture of clinical characteristics and outcomes, the retrospective design relied on clinical records, which often lacked key details, including symptom onset, vaccination status, antiviral timing, and escalation‐of‐care criteria. Residual confounding may arise from unmeasured factors such as healthcare‐seeking behavior, clinician decision‐making, and comorbidity severity. Outcomes such as ICU admission and respiratory support, although commonly used as markers of severity, may also reflect differences in institutional practices and resource availability rather than disease severity alone. Influenza B lineage data were not available in the EMR preventing assessment of lineage‐specific severity, though B/Yamagata has not been detected in Australia since 2020. Influenza vaccination status could not be ascertained, as it is not routinely recorded in the eMR or linked to Australian Immunization Register. Vaccination status is an important potential confounder, particularly given age‐related differences in vaccine eligibility, funding, and uptake in Australia. Influenza vaccine coverage in children has historically been suboptimal, especially in older age groups. As children with influenza B in our cohort were older, lower vaccine uptake may have contributed to increased severity. Therefore, the observed associations between influenza B and higher severity markers should not be interpreted as evidence of greater intrinsic virulence, but rather as findings that may be influenced by differential vaccine protection. The 2‐year post‐pandemic study period may also limit generalizability due to altered viral circulation and healthcare practices. The biennial fluctuating prevalence of influenza B further highlights the need for longer observation periods to assess its full impact. Incorporating standardized diagnostic tools could strengthen data quality, although qualitative research into clinical decision‐making may help clarify variations in respiratory support practices. Testing practices during the post‐COVID period may also have influenced case ascertainment. Changes in respiratory virus testing strategies, including broader use of multiplex PCR testing, may have altered the identification of influenza cases over time. These factors should be considered when interpreting our findings within the broader context of evolving healthcare practices. Also, our study does not include a pre‐pandemic comparator group. As such, we were unable to determine whether the relative severity patterns between influenza A and B have changed as a result of COVID‐19‐related disruptions. Instead, our findings should be interpreted as a description of influenza‐related hospitalizations in a post‐pandemic healthcare context. In conclusion, in this cohort of hospitalized children in the post‐COVID era, influenza B was associated with higher markers of in‐hospital severity compared with influenza A. However, these findings should be interpreted cautiously given potential confounding by age, vaccination status, and admission practices.

## 5 Conclusions

This study highlights the significant burden of influenza on Australian children, with influenza B associated with greater illness severity, particularly in older age groups. Our findings support the continued inclusion of circulating B lineages in pediatric influenza vaccines. Future prospective studies incorporating immunization data and pre‐pandemic comparisons are needed to better understand subtype‐specific disease severity in children and inform future policy considerations, including expanding NIP eligibility to all children aged ≥ 6 months.

## Author Contributions

Nusrat Homaira, Adam Jaffe, Mei Chan, Philip N. Britton, Adam W. Bartlett, Rama Kandasamy, Louisa Owens, and Bernadette Prentice conceptualized and designed the study and provided critical feedback in drafting the manuscript. Angelica Wei Wen Hii conducted the study, analyzed the data, and drafted the manuscript. Jahidur Rahman Khan and Kishor Kumar Paul provided overall supervision in the conduct of the study and provided critical feedback in drafting the manuscript. Nan Hu provided technical assistance with statistical analysis of the study and provided critical feedback in drafting the manuscript. All authors approved the final manuscript as submitted and agree to be accountable for all aspects of the work.

## Funding

The authors have nothing to report.

## Ethics Statement

Ethics approval was granted by the SCHN Human Research Ethics Committee (HREC reference number 2022/ETH01866).

## Conflicts of Interest

Nusrat Homaira and Rama Kandasamy at times have received consultation fees from Sanofi, Pfizer, Seqirus, and Merck Sharp & Dohme Australia. Nusrat Homaira, Rama Kandasamy, Philip N. Britton, and Adam W. Bartlett are chief investigators of industry‐sponsored studies. The other authors declare no conflicts of interest.

## Supporting information


**Table S1:** Clinical presentation at admission by age group and influenza type among children < 18 years hospitalized with influenza A or B at Sydney Children's Hospital Network, 2022–2023*.

## Data Availability

The data that support the findings of this study are available on request from the corresponding author. The data are not publicly available due to privacy or ethical restrictions.
