# Supplementary material for: In‐Hospital Complications of Influenza A and B Among Hospitalized Australian Children in the Post‐COVID Era
Source: Clin Respir J. 2026 Jul 23;20(7):e70217. doi: 10.1111/crj.70217 (PMC13393287; doi:10.1111/crj.70217)
Supplement: Supplementary file 1 — Table S1: Clinical presentation at admission by age group and influenza type among children < 18 years hospitalized with influenza A or B at Sydney Children's Hospital Network, 2022–2023*. [file CRJ-20-e70217-s001.docx]

**Supplementary Table 1.** Clinical Presentation at Admission by Age Group and Influenza Type Among Children <18 Years Hospitalized with Influenza A or B at Sydney Children’s Hospital Network, 2022–2023*

| **Clinical Presentation at Admission** | **0–<5 years** | | **5–<18 years** | | **All Age Groups** | |
| --- | --- | --- | --- | --- | --- | --- |
|  | **Influenza A (n=203)** | **Influenza B (n=73)** | **Influenza A (n=277)** | **Influenza B (n=151)** | **Influenza A (n=480)** | **Influenza B (n=224)** |
| Apnea | 1 (0.5) | 2 (2.7) | 2 (0.7) | 0 | 3 (0.6) | 2 (0.9) |
| Central cyanosis | 5 (2.5) | 1 (1.4) | 5 (1.8) | 0 | 10 (2.1) | 1 (0.4) |
| Chest pain | 2 (1.0) | 1 (1.4) | 24 (8.7) | 9 (6.0) | 26 (5.4) | 10 (4.5) |
| Conjunctivitis | 10 (4.9) | 2 (2.7) | 13 (4.7) | 2 (1.3) | 23 (4.8) | 4 (1.8) |
| Cough | 178 (87.7) | 59 (80.8) | 236 (85.2) | 114 (75.5) | 414 (86.3) | 173 (77.2) |
| Crackles | 59 (29.1) | 18 (24.7) | 30 (10.8) | 15 (9.9) | 89 (18.5) | 33 (14.7) |
| Dyspnea | 13 (6.4) | 7 (9.6) | 32 (11.6) | 18 (11.9) | 45 (9.4) | 25 (11.2) |
| Fever | 188 (92.6) | 68 (93.2) | 264 (95.3) | 141 (93.4) | 452 (94.2) | 209 (93.3) |
| Gastrointestinal symptoms | 123 (60.6) | 52 (71.2) | 198 (71.5) | 113 (74.8) | 321 (66.9) | 165 (73.7) |
| Headache | 9 (4.4) | 3 (4.1) | 95 (34.3) | 49 (32.5) | 104 (21.7) | 52 (23.2) |
| Hypoxia | 48 (23.6) | 16 (21.9) | 40 (14.4) | 18 (11.9) | 88 (18.3) | 34 (15.2) |
| Lethargy | 128 (63.1) | 54 (74.0) | 153 (55.2) | 79 (52.3) | 281 (58.5) | 133 (59.4) |
| Myalgia | 11 (5.4) | 5 (6.8) | 63 (22.7) | 54 (35.8) | 74 (15.4) | 59 (26.3) |
| Nasal symptoms | 166 (81.8) | 57 (78.1) | 176 (63.5) | 98 (64.9) | 342 (71.3) | 155 (69.2) |
| Pharyngitis | 22 (10.8) | 25 (34.2) | 109 (39.4) | 67 (44.4) | 131 (27.3) | 92 (41.1) |
| Reduced oral intake | 162 (79.8) | 61 (83.6) | 192 (69.3) | 102 (67.5) | 354 (73.8) | 163 (72.8) |
| Seizures | 22 (10.8) | 4 (5.5) | 13 (4.7) | 1 (0.7) | 35 (7.3) | 5 (2.2) |
| Severe respiratory distress | 34 (16.7) | 15 (20.5) | 15 (5.4) | 19 (12.6) | 49 (10.2) | 34 (15.2) |
| Tachypnoea | 43 (21.2) | 11 (15.1) | 51 (18.4) | 21 (13.9) | 94 (19.6) | 32 (14.3) |
| Wheeze | 36 (17.7) | 10 (13.7) | 35 (12.6) | 9 (6.0) | 71 (14.8) | 19 (8.5) |

* Data are presented as n (column %) unless otherwise specified.
